# Content validation of the Qualipreterm: quality of preterm infant follow-up in Primary Health Care

**DOI:** 10.1590/0034-7167-2024-0123

**Published:** 2025-06-30

**Authors:** Sheila Rodrigues Paião, Adriana Zilly, Marcos Augusto Moraes Arcoverde, Débora Falleiros de Mello, Rosane Meire Munhak da Silva

**Affiliations:** I Universidade Estadual do Oeste do Paraná. Foz do Iguaçu, Paraná, Brazil.; II Universidade de São Paulo, Ribeirão Preto, São Paulo, Brazil.

**Keywords:** Infant, Premature, Continuity of Patient Care, Primary Health Care, Validation Study, Quality of Health Care, Recién Nacido Prematuro, Continuidad de la Atención al Paciente, Atención Primaria de Salud, Estudio de Validación, Calidad de la Atención Sanitaria

## Abstract

**Objectives:**

To validate the content of the Qualipreterm guide, which assesses the quality of follow-up care for preterm infants in Primary Health Care.

**Methods:**

We conducted a methodological study using the Delphi technique. In the first round, we included 17 judges (nurses, master’s degree holders, and doctoral experts in neonatal health). In the second round, we included 20 nurses actively working in Primary Health Care. We performed data analysis using Cronbach’s alpha, with a minimum acceptable value of 0.70.

**Results:**

During the content validation phase, the overall Cronbach’s alpha was 0.989, and across the domains, it exceeded 0.957. For the “Clarity” and “Comprehensibility” dimension, Cronbach’s alpha was 0.993 in the second round, demonstrating satisfactory agreement among the items. All alpha coefficients surpassed 0.876 when distributed across the domains.

**Conclusions:**

The evaluative Qualipreterm guide’s content achieved validation criteria and incorporated the received recommendations. In the final version, it contained 64 items.

## RESUMEN

**Objetivo:**

Validar el contenido del guía Qualiprematuro para evaluar la calidad del seguimiento de niños prematuros en la Atención Primaria de Salud.

**Métodos:**

Estudio metodológico basado en la Técnica Delphi, con 17 jueces (enfermeros, magísteres y doctores expertos en salud neonatal) en la primera etapa y 20 enfermeros de la Atención Primaria en la segunda. El análisis utilizó el coeficiente alfa de Cronbach, con un valor mínimo aceptable de 0,70.

**Resultados:**

En la validación de contenido, el coeficiente alfa general fue 0,989, con valores superiores a 0,957 en los dominios. En “Claridad” y “Comprensibilidad,” el coeficiente fue 0,993, mostrando concordancia satisfactoria, y todos los coeficientes superaron 0,876 en la distribución entre los dominios.

**Conclusión:**

El contenido del Qualiprematuro fue validado, incorporó las recomendaciones de los evaluadores y finalizó con 64 preguntas.

## INTRODUCTION

Preterm birth is considered a global public health issue. Each year, approximately 15 million babies are born preterm, with 1.1 million deaths occurring annually, of which 75% are deemed preventable^(1)^.

The prevalence of preterm births is higher in low-income countries, with 60% concentrated in South Asia and Sub-Saharan Africa. In the poorest nations, more than 12% of babies are born preterm, compared to 9% in higher-income countries. It is essential to highlight that this issue is not limited to low-income nations, as both the United States and Brazil rank among the top ten countries with the highest number of preterm births^(1-3)^.

Preterm birth and low birth weight are directly linked to neonatal mortality, representing a significant challenge for healthcare systems and emphasizing the need for greater integration between prenatal and neonatal care services^(4)^.

During hospitalization, the development of complications emerges as a critical factor, increasing both the risk of mortality and the likelihood of worsening clinical conditions in preterm infants. Additionally, adverse situations stemming from these complications, therapeutic interventions, and healthcare processes can result in a higher number of children requiring complex care, with implications for their long-term health follow-up^(4)^.

Despite the recognized progress in healthcare aimed at improving the survival of infants in Neonatal Intensive Care Units (NICUs), it is important to note that the establishment of neonatal care units alone does not reduce morbidity and mortality rates. Successful treatment is not determined solely by survival and hospital discharge but also by building bonds and implementing actions that ensure continuity of care after discharge^(5,6)^.

Primary Health Care (PHC) plays a crucial role in coordinating care within the broader Healthcare Network^(7)^. The effectiveness of this network depends on: providing comprehensive and clear health actions for surveillance, disease prevention, and health promotion; maintaining care continuity through regulated access and integration with other levels of care; employing well-trained generalist professionals who utilize the best scientific evidence; offering a wide range of services; and fostering matrix-based integration with specialists^(7)^.

The involvement of PHC professionals is essential to ensure quality follow-up care for preterm newborns, promoting comprehensive care after hospital discharge, given their potential vulnerabilities, illnesses, and growth and developmental challenges^(8)^. However, despite advances in longitudinal care within PHC, weaknesses persist regarding the necessary care for preventable and sensitive causes of health monitoring^(9)^.

It is, therefore, necessary to assess PHC services in terms of their practices to ensure continuity of care for preterm infants. In this context, given the importance of the topic, this study aimed to validate the content of the “Qualipreterm” guide (originally “Qualiprematuro” in Portuguese)^(10)^, developed to assess the quality of healthcare follow-up for preterm children within PHC. The guide was created in 2021 based on postdoctoral nursing and public health research. Its development was justified by findings from studies identifying gaps in healthcare follow-up for preterm infants within PHC in Brazil, particularly in regions with heightened vulnerabilities, such as the tri-border area — Brazil, Paraguay, and Argentina^(4,11,12)^.

The need to evaluate actions related to preterm follow-up in PHC stems from the understanding that these services must provide comprehensive, appropriate, timely, and evidence-based practices. The ultimate goal is for such services to improve living and health conditions and ensure optimal child development, especially for preterm infants

## OBJECTIVE

This study aims to validate the content of the Qualipreterm guide for evaluating the quality of follow-up care provided to preterm infants within Primary Health Care (PHC).

## METHODS

### Ethical aspects

The research received approval from the Research Ethics Committee and adhered to all ethical guidelines for research involving human subjects, as outlined in Resolution No. 466/2012. To ensure participant anonymity, judges were identified using the letter J followed by a sequential evaluation number (e.g., J1, J2). PHC nurses who served as judges were identified with the letter N followed by the evaluation number (e.g., N1, N2). All participants provided informed consent, with most consenting online.

### Study design

This methodological study employed the Delphi Technique^(13)^ to validate the content of the Qualipreterm guide^(10)^. The study followed the Standards for Quality Improvement Reporting Excellence (SQUIRE) guidelines^(14)^, designed for descriptive reports on studies to improve healthcare quality.

#### Description of the Qualipreterm guide

The first version of the Qualipreterm guide^(10)^ was developed in 2021 and included a 65-item questionnaire to be completed by healthcare professionals (nurses or physicians) working in PHC services. The guide is organized into five domains to encompass elements critical for quality follow-up care of preterm infants: Hospital discharge planning and care plan organization (11 questions); Home follow-up through visits and telehealth (11 questions); Child health follow-up to promote health and prevent complications (12 questions); Integration between health services, education, and specialized care (19 questions); and Family support and care assistance (12 questions).

Each question offered four single-choice responses, scored as follows: 1 (Inadequate), 2 (Regular), 3 (Good), and 4 (Excellent). At the end of each domain’s evaluation, scores were summed to indicate how effectively the health service follows up with preterm infants. The guide allows for the separate evaluation of each domain, recognizing that a service might score inadequately in one domain while excelling in another. Based on the total scores for each domain, services were categorized as Inadequate, Regular, Adequate/Good, or Excellent.

#### Study location and participant selection

We conducted the study in two phases. In the first phase, we contacted online judges from various Brazilian states. In the second phase, the selected judges were nurses working in PHC services in the municipality of Foz do Iguaçu, Paraná state (PR), Brazil.

To identify judges for the first phase, we utilized search filters on the Lattes Platform (a Brazilian virtual system of academic curricula) of the National Council for Scientific and Technological Development (CNPq). We conducted additional searches on the websites of stricto sensu postgraduate programs at Brazilian public universities.

In the first validation phase, the 17 judges included were nurses with a minimum academic qualification of a master’s degree (6 with master’s degrees and 11 with doctoral degrees). These professionals worked directly with newborns and/or conducted research in neonatal and child health, as indicated in their Lattes profiles. We excluded nurses who did not respond to the invitation after three contact attempts (via email or instant messaging). We initially sent 50 invitations to professionals from all regions of Brazil, with responses from 17 professionals, all from the South and Southeast regions, who proceeded to evaluate the guide’s content.

In the second phase, researchers selected 20 judges who were nurses actively working in PHC services in a Brazilian border municipality. The exclusion criterion for this phase was being on leave or vacation during the data collection period.

We determined the sample for the content validation process across both phases based on methodologies used in similar instrument validation studies^(15,16)^.

#### Data collection

We initiated the first round of questionnaire distribution to the judges in June 2023. A subsequent distribution occurred in August 2023 to ensure a representative number of participants. The second stage of data collection took place in September 2023. Additional rounds of evaluation were unnecessary after the statistical analyses using the Delphi Technique, as the consensus level proposed by the method was achieved in the first round.

We invited judges from the first stage through an invitation letter sent via email and/or WhatsApp^®^. The message outlined the study’s objectives and explained that participants who accepted the invitation were required to digitally sign the Informed Consent Form (ICF) in duplicate—one copy remained with the judge, and the other was returned to us for archival purposes. After consent, we sent the guide for evaluation and validation via email and/or Google Forms^®^. Once reviewed, judges returned the guide online with recommendations for modifications.

In the second stage, participating nurses represented health units across the five districts of Foz do Iguaçu, Paraná. This distribution allowed us to assess the clarity and comprehensibility of the guide across the entire municipality. We selected the health units through random sampling. Subsequently, we extended invitations to nurses via phone calls and/or text messages. All 20 nurses in this stage signed the ICF online using Google Forms^®^.

#### Data collection instrument

For the first stage, we used an adapted version of the Qualipreterm guide as the data collection instrument. Participants were asked to rate each question using the following interpretations: “Agree,” “Partially Agree,” and “Disagree.” These interpretations were organized into a Likert Scale, assigning values as follows: (1) Disagree, (2) Partially Agree, and (3) Agree. If a judge selected “Disagree” or “Partially Agree,” a text box was provided to suggest revisions for the question.

In the second stage, nurses evaluated each question using the interpretations: “Confusing,” “Unclear,” and “Clear and Easy to Understand.” These were similarly organized into a Likert Scale with the following values: (1) Confusing, (2) Unclear, and (3) Clear and Easy to Understand. Space was also provided for nurses to offer suggestions if they rated a question as “Confusing” or “Unclear.”

#### Data analysis

We analyzed the results using Cronbach’s alpha coefficient, with a minimum acceptable value of 0.70. Values below this cutoff point indicated inconsistency or inadequacy in the questions. Conversely, values exceeding 0.90 could signal redundancy or duplication among the evaluated items within the same element or construct. Ideally, alpha values between 0.80 and 0.90 were preferred in such cases^(17)^.

### RESULTS

#### Characterization of judges participating in the first phase

Seventeen judges participated in the first phase. All were nurses with a minimum academic qualification of a master’s degree, working in teaching positions at public universities and/or practicing professionally with premature children in Brazil’s South and Southeast regions.

#### Reliability analysis of the Qualipreterm guide – first phase

For the internal consistency analysis of the guide (reliability analysis), we excluded Questions 30, 40, 43, 46, 47, 50, 56, 58, 64, and 65 because all 17 participants provided the same response (“Agree”).

Based on Cronbach’s alpha coefficient, the overall reliability analysis of the guide demonstrated satisfactory correlation among the questions, with a value of 0.989. When analyzed separately, all five domains also showed satisfactory reliability, as presented in Table 1.

**Table 1:** Reliability analysis distributed across the domains of the Qualipreterm guide. Foz do Iguaçu, Paraná, Brazil, 2023

Scales	Cronbach’s alpha
Total analysis	0.989
Domain I	0.970
Domain II	0.957
Domain III	0.958
Domain IV	0.972
Domain V	0.965

The correlation analysis indicated that even if any question were removed, Cronbach’s alpha would remain between 0.989 and 0.990, which are highly satisfactory values.

To refine the guide evaluation, we analyzed the test value for each question, verifying the potential need to remove any question based on “item-total correlation” values, which measure the correlation of the total test score with each individual question. According to the assessment, while no statistical necessity for removal was identified, we excluded Question 6 from Domain I based on the judges’ qualitative judgment due to redundancy. For this question, Cronbach’s alpha item-total correlation analysis yielded satisfactory results, ranging from 0.948 to 0.974.

#### Characterization of primary health care nurses – second phase

Twenty nurses participated in the phase assessing the clarity and comprehensibility of the guide. Among them, 3 were men, and 17 were women, aged between 29 and 47 years. The group included 5 individuals with master’s degrees, 2 master’s students, and 13 specialists in various health fields. Most participants had been working in PHC for over five years.

#### Reliability analysis of the Qualipreterm guide: clarity and comprehensibility

Based on Cronbach’s alpha, the overall reliability analysis showed a satisfactory consensus level, with an upper scale value of 0.993. The domain-specific reliability analysis also demonstrated satisfactory correlation coefficients, although a decrease was noted in Domain III without compromising overall reliability, as detailed in Table 2.

**Table 2:** Reliability analysis regarding the clarity and comprehensibility of the Qualipreterm guide, distributed across its domains. Foz do Iguaçu, Paraná, Brazil, 2023

Scales	Cronbach’s alpha
Total analysis	0.993
Domain I	0.961
Domain II	0.977
Domain III	0.876
Domain IV	0.984
Domain V	0.981

In the item-total correlation analysis, Cronbach’s alpha yielded satisfactory results, ranging from 0.850 to 0.985. However, Questions 11, 21, 23, 46, and 63 showed weaker item-total correlations. Even so, removing any of these questions would not negatively affect the reliability analysis, as illustrated in Table 3. Therefore, we decided to retain these questions, as they address critical aspects of premature child follow-up.

**Table 3:** Reliability statistics of questions with low item-total correlation distributed across the five domains. Foz do Iguaçu, Paraná, Brazil, 2023

	If the item is removed	
	Item-total correlation	Cronbach’s alpha
Q11	0.513	0.965
Q21	0.688	0.980
Q23	0.196	0.883
Q46	0.546	0.985
Q63	0.667	0.984

Considering the judges’ qualitative analysis, which recommended the removal of Question 6 from Domain I due to redundancy, the Qualipreterm guide required adjustments. These adjustments involved renumbering all questions across domains and recalculating the evaluation score for Domain I, which initially ranged from 11 to 44 and was revised to a range of 10 to 40. Table 1 outlines these adjustments.

### DISCUSSION

This study provided a detailed perspective on the content validation process of the Qualipreterm guide, whose primary objective is to assess the quality of follow-up care for preterm children within PHC. This valuable tool in the healthcare field underwent a thorough evaluation by judges who are researchers and/or experienced professionals in neonatal and child health. Their extensive background ensured a precise and specialized assessment of the guide. Additionally, healthcare professionals (nurses) working in PHC from a Brazilian municipality in a tri-border area—Brazil, Paraguay, and Argentina—analyzed the clarity of its content.

Involving these judges emphasized the importance of considering local realities when assessing the effectiveness of preterm infant follow-up within PHC, thereby ensuring the guide’s applicability and efficacy^(15,16)^. This approach aimed to guarantee a significant representation of the knowledge and experience required to evaluate the Qualipreterm guide.

The participation of PHC professionals was particularly valuable in enhancing the clarity of the guide. This ensures comprehensive care following hospital discharge, considering the increased risk of illness and potential developmental and growth challenges^(7,12)^. Notably, despite advancements in longitudinal care within PHC, persistent weaknesses remain in addressing preventable causes of mortality and health challenges, particularly among vulnerable groups such as preterm children.

The content validation analysis of the Qualipreterm guide aimed to refine the tool and provide practical support for improving the effectiveness of PHC services^(10)^. Including border areas in the analysis highlighted the need to account for diverse geographic contexts when planning interventions for preterm infants, thereby promoting a context-specific approach^(4,11)^. This strategy sought to enhance the validity and reliability of the results, contributing to the methodological quality of this evaluative guide.

The choice of the Delphi Technique was instrumental in developing a robust and reliable guide. This technique, recognized as a tool for structuring and validating information through expert consensus^(18)^, was critical in evaluating items related to the quality of health surveillance for preterm infants in PHC, addressing a gap in the scientific literature on assessing these specific aspects of care^(10)^. Conducting two rounds of evaluation demonstrated a meticulous methodology, highlighting the consistency and representativeness of the collected data.

Using a Likert scale for data collection, with responses ranging from “Disagree” to “Agree,” enabled a quantitative approach essential for subsequent statistical analysis. This technique is widely applied in social studies to collect non-quantitative perceptions on specific topics^(19)^. Additionally, incorporating spaces for reformulation suggestions introduced a qualitative dimension, allowing judges to provide detailed opinions on each item in the guide.

We analyzed the results using Cronbach’s alpha coefficient^(20)^, which demonstrated an overall satisfactory score in the evaluations of both judge groups (researchers/professionals and PHC nurses), with values of 0.989 and 0.993, respectively. Similarly, we observed satisfactory results across the five domains evaluated. It is crucial to recognize that the number of items in the scale influences the alpha value. As the number of items increases, variance is systematically incorporated into the numerator, leading to inflated estimates of scale consistency^(21,22)^. Furthermore, it is essential to consider that ignoring sample size can also inflate Cronbach’s alpha values^(23,24)^.

Studies comparing validation instruments using Cronbach’s alpha have demonstrated that the intensity of correlation between questionnaire items can be evaluated by removing an item from the measurement scale, as performed in this study, with equally satisfactory results regardless of the item removed^(25,26)^. If the alpha coefficient increases, the removed item likely lacks a strong correlation with the remaining items. Conversely, a decrease in the coefficient suggests the item is highly correlated with others in the scale. Thus, Cronbach’s alpha served as a crucial indicator for assessing the guide’s reliability, revealing how each item contributed to this reliability, with values ranging from 0.949 to 0.976 for the judges and from 0.850 to 0.985 for PHC nurses.

The proposed guide comprehensively evaluated preterm child health and was initially composed of 65 items. Following qualitative feedback from participants, one item from Domain I was removed due to redundancy. The guide was subsequently reorganized into 64 items, all designed to uncover key nuances of care provided by professionals—primarily physicians and nurses—and the attention delivered through PHC^(10)^.

The five-domain structure remained unchanged, encompassing hospital discharge planning, home follow-up, child health, service integration, and family support. This organization ensured comprehensive coverage of preterm child health follow-up conditions. These diverse areas reflect the complexity of supporting preterm children, from discharge planning to essential family support. The guide’s question-specific responses aimed to provide a detailed, evidence-based evaluation of the effectiveness of follow-up care^(10,27)^.

Since this study conducted content validation steps, summing values at the end of each proposed domain provided a critical metric for analyzing the care delivered. Evaluating each area individually while recognizing that a service may excel in some domains but require improvement in others encourages healthcare teams and services to strive for better practices and service organization.

Content validation ensures an instrument measures what it claims to measure^(28)^. This is fundamental for producing reliable results that can safely inform decision-making. Validation helps identify and rectify potential tool deficiencies, ensuring precision in responses, which is especially critical when dealing with sensitive information that may have significant consequences.

It is essential to emphasize that validity is intrinsically linked to an instrument’s reliability. In other words, validity can be understood as the degree of association between test scores and an external criterion of the same test, such as results from another evaluation, conceptual definitions, or formulated objectives^(29,30)^.

The validation process establishes the initial reliability of the tool and provides insights for its ongoing improvement. Assessing the quality of preterm child health follow-up benefits these individuals and enhances the efficiency and effectiveness of PHC services.

Currently, literature on preterm child care and follow-up remains limited. The proposal to validate the Qualipreterm guide’s content, focusing on a border region, evaluates a specific cultural context and reinforces the importance of tools that support and assess follow-up health actions and practices.

This study provided a critical perspective on current practices, highlighting potential areas for improvement in addressing preterm child health within PHC. The focus on border regions, where PHC nurses in this study worked, acknowledges the vulnerabilities of these territories and aims to optimize health follow-up strategies^(4,11,12)^.

Additionally, the online Delphi Technique enabled the participation of judges from various locations, ensuring diverse experiences contributed to validating the guide. Addressing specific issues across a real regional diversity underscores the importance of considering unique geographic contexts when developing surveillance and monitoring strategies. Data analysis was iterative and achieved a consistent value as proposed by the statistical method. These results are expected to inspire future initiatives and encourage research to monitor preterm infant health, providing a solid foundation for timely and effective interventions.

Investing in the health of preterm children should extend beyond hospitalization, as each child holds the potential to overcome early life challenges and become a healthy, contributing member of society. Therefore, ensuring the health of preterm children promotes a healthy start and represents a significant commitment to building a safe and sustainable community^(1,2)^.

### Study limitations

In the content validation process of the Qualipreterm guide, we did not perform a pretest or pilot study phase, which should be addressed in future research. Additionally, it will be relevant to include other PHC professionals involved in the follow-up of preterm children, as this study focused exclusively on nurses.

### Contributions to the field of nursing

The content validation of the Qualipreterm guide provided valuable, standardized, and culturally sensitive data. As such, it becomes an appropriate tool for training healthcare professionals, particularly nurses, in delivering effective and personalized support to these children and their families. The contributions extend beyond measuring outcomes, thus influencing nursing practices in research and promoting health policies aimed at the global development and well-being of preterm children.

By providing insights into the quality of surveillance for preterm infants, we aim to broaden the understanding of healthcare practices in Primary Health Care, particularly in vulnerable regions such as border areas. This contribution aligns with the Sustainable Development Goals and the Global Strategy for Women’s, Children’s, and Adolescents’ Health, emphasizing the adoption of more integrated and collaborative approaches to address social, environmental, and economic inequalities.

The content of the Qualipreterm guide improves the quality of preterm child health follow-up by ensuring that these children receive appropriate attention and care in PHC. Furthermore, it standardizes and guides clinical practice with validated recommendations and guidelines. Additionally, it can enhance team communication and the effectiveness of healthcare services, thereby benefiting the coordination of care for preterm children

## CONCLUSION

We validated the content of the Qualipreterm guide, demonstrating its representativeness in the theoretical and practical understanding of care. This validation process marked a crucial phase in the guide’s development, confirming the material’s reliability and conciseness in measuring its intended constructs.

Utilizing the Delphi Technique, our methodology facilitated consensus among experts—judges with neonatal health and research expertise—and nurses with professional experience in PHC. The iterative nature of data analysis, supported by satisfactory Cronbach’s alpha scores across the two evaluation phases and the domain-level distribution analysis, ensured the results’ reliability, validity, and clarity. Importantly, even with favorable indices, we incorporated all recommendations from the specialists into the guide’s final construction. Initially, Domain I included 11 questions, which were refined to 10 after adjustments. Overall, the initial version comprised 65 questions, while the final version contained 64 questions.

Thus, the content validation of the Qualipreterm guide, established as an innovative evaluation tool with a comprehensive methodology, will contribute to the theoretical understanding of care and recommend critical practices for healthcare professionals in the follow-up of preterm child health. The study’s commitment to transparency, consistency, and methodological rigor strengthens future initiatives and research focused on the health surveillance of preterm children, addressing the pressing need to improve healthcare practices and outcomes for this population worldwide.

**Chart 1:** Distribution of questions across the five domains after the adjustment of the Qualipreterm guide and their respective evaluation scores. Foz do Iguaçu, Paraná, Brazil, 2023

Domains	Previous questions	Current questions	Scores from the first evaluation	Scores from the final evaluation
I	1,2,3,4,5, 6,7,8,9,10, 11	1,2,3,4,5, 6,7,8,9,10	*Inadequate* (score 11 to 21) *Regular* (score 22 to 32) *Good* (score 33 to 43) *Excellent* (score 44)	*Inadequate* (score 10 to 19) *Regular* (score 20 to 29) *Good* (score 30 to 39) *Excellent* (score 40)
II	12,13,14, 15,16,17, 18,19,20, 21,22	11,12,13, 14,15,16, 17,18,19, 20,21	*Inadequate* (score 11 to 21) *Regular* (score 22 to 32) *Good* (score 33 to 43) *Excellent* (score 44)	*Inadequate* (score 11 to 21) *Regular* (score 22 to 32) *Good* (score 33 to 43) *Excellent* (score 44)
III	23,24,25, 26,27,28, 29,30,31, 32,33,34	22,23,24, 25,26,27, 28,29,30, 31,32,33	*Inadequate* (score 12 to 23) *Regular* (score 24 to 35) *Good* (score 36 to 47) *Excellent* (score 48)	*Inadequate* (score 12 to 23) *Regular* (score 24 to 35) *Good* (score 36 to 47) *Excellent* (score 48)
IV	35,36,37, 38,39,40, 41,42,43, 44,45,46, 47,48,49, 50,51,52, 53	34,35,36, 37,38,39, 40,41,42, 43,44,45, 46,47,48, 49,50,51, 52	*Inadequate* (score 19 to 37) *Regular* (score 38 to 56) *Good* (score 57 to 75) *Excellent* (score 76)	*Inadequate* (score 19 to 37) *Regular* (score 38 to 56) *Good* (score 57 to 75) *Excellent* (score 76)
V	54,55,56, 57,58,59, 60,61,62, 63,64,65	53,54,55, 56,57,58, 59,60,61, 62,63,64	*Inadequate* (score 12 to 23) *Regular* (score 24 to 35) *Good* (score 36 to 47) *Excellent* (score 48)	*Inadequate* (score 12 to 23) *Regular* (score 24 to 35) *Good* (score 36 to 47) *Excellent* (score 48)
