# Supplementary material for: Content validation of the Qualipreterm: quality of preterm infant follow-up in Primary Health Care
Source: Rev Bras Enferm. 2025 Jun 30;78(2):e20240123. doi: 10.1590/0034-7167-2024-0123 (PMC12221271; doi:10.1590/0034-7167-2024-0123)
Supplement: Translation

# Translation: Validação de conteúdo do Qualiprematuro: qualidade do seguimento de prematuros na Atenção Primária à Saúde

**DOI:** 10.1590/0034-7167-2024-0123pt

**Authors:** Sheila Rodrigues Paião, Adriana Zilly, Marcos Augusto Moraes Arcoverde, Débora Falleiros de Mello, Rosane Meire Munhak da Silva

**Affiliations:** I Universidade Estadual do Oeste do Paraná. Foz do Iguaçu, Paraná, Brazil.; II Universidade de São Paulo, Ribeirão Preto, São Paulo, Brazil.

**Keywords:** Recém-Nascido Prematuro, Continuidade da Assistência ao Paciente, Atenção Primária à Saúde, Estudo de Validação, Qualidade da Assistência à Saúde

## Abstract

**Objetivos:**

Validar o conteúdo do guia Qualiprematuro sobre avaliação da qualidade do seguimento de crianças nascidas prematuras no âmbito da Atenção Primária à Saúde.

**Métodos:**

Estudo metodológico, baseado na Técnica Delphi, com a participação de 17 juízes (enfermeiros, mestres e doutores experts em saúde neonatal) na primeira etapa e 20 profissionais atuantes na Atenção Primária à Saúde (enfermeiros) na segunda. Realizou-se análise pelo coeficiente alfa de Cronbach, com valor mínimo aceitável 0,70.

**Resultados:**

Na etapa de validação de conteúdo, o coeficiente geral alfa de Cronbach foi de 0,989 e entre os domínios foi acima de 0,957. Para “Clareza” e “Compreensibilidade”, o coeficiente alfa de Cronbach foi 0,993 na segunda etapa, mostrando concordância satisfatória das questões, e todos os coeficientes alfa foram acima de 0,876 na distribuição entre os domínios.

**Conclusões:**

O conteúdo do guia avaliativo Qualiprematuro foi considerado validado, incorporou recomendações recebidas e, ao final, compreendeu 64 questões.

## INTRODUÇÃO

A prematuridade é considerada um problema de saúde pública mundial. Entre os nascimentos, cerca de 15 milhões de bebês nascem prematuros, com ocorrência de 1,1 milhão de mortes a cada ano, das quais 75% são consideradas evitáveis^(1)^.

O nascimento prematuro tem maior ocorrência em países de baixa renda, visto que 60% concentram-se no sul da Ásia e na África Subsaariana. Nos países mais pobres, mais de 12% dos bebês nascem prematuros, em comparação a 9% em países de maior renda. É importante destacar que esse problema não está limitado apenas aos países de baixa renda, pois os Estados Unidos e o Brasil encontram-se entre os dez com o maior número de partos prematuros^(1-3)^.

A prematuridade e o baixo peso ao nascer estão diretamente associados a mortalidade no período neonatal. Esse fato representa um desafio significativo para os sistemas de saúde e indica a necessidade de maior integração dos serviços de pré-natal e de atenção neonatal^(4)^.

Ao longo da hospitalização, o desenvolvimento de complicações emerge como um fator crucial e eleva tanto o risco de mortalidade quanto a possibilidade de agravamentos das condições clínicas de crianças nascidas prematuras. Além disso, situações adversas decorrentes dessas complicações, das intervenções terapêuticas e dos processos de trabalho podem resultar em maior número de crianças que necessitam de cuidados complexos, com repercussões no seguimento de sua saúde^(4)^.

Apesar de serem amplamente reconhecidos os progressos da atenção à saúde para a sobrevida dos bebês em Unidades de Terapia Intensiva Neonatal (UTIN), é importante destacar que a implementação apenas de unidades de cuidados neonatais não resulta na redução da morbimortalidade. O sucesso do tratamento não é determinado somente pela sobrevivência e alta hospitalar, mas também pela construção de vínculos e ações que garantirão a continuidade dos cuidados pós-alta^(5,6)^.

A Atenção Primária à Saúde (APS) é fundamental, pois coordena o cuidado nos pontos da Rede de Atenção à Saúde^(7)^. O funcionamento da rede depende de: garantia de ações de saúde abrangentes e claras para vigilância, prevenção de doenças e promoção da saúde; continuidade da gestão do cuidado por meio do acesso regulado e integração com outros níveis de atenção; profissionais generalistas treinados, utilizando as melhores evidências científicas; ampla gama de cuidados; e integração matricial com especialistas^(7)^.

O envolvimento da equipe profissional da APS torna-se crucial para assegurar o seguimento de qualidade aos recém-nascidos prematuros, garantindo a atenção integral após a alta hospitalar, visto que eles podem apresentar vulnerabilidades, adoecimentos e alterações em seu crescimento e desenvolvimento^(8)^. Contudo, embora haja avanços no que se refere ao cuidado longitudinal na APS, ainda existem fragilidades relacionadas ao cuidado necessário diante das causas evitáveis e sensíveis de acompanhamento de saúde^(9)^.

Portanto, é preciso avaliar os serviços da APS no tocante às práticas que envolvem a continuidade do cuidado em saúde às crianças nascidas prematuras. Dessa forma, pela importância do tema, o presente estudo pretendeu validar o conteúdo do guia denominado “Qualiprematuro”^(10)^, organizado para avaliar a qualidade do seguimento da saúde da criança prematura no âmbito da APS. O referido guia foi elaborado em 2021, com base em uma pesquisa de pós-doutorado na área de enfermagem em saúde pública. Sua construção teve como justificativa os resultados de estudos que identificaram fragilidades no seguimento da saúde da criança prematura no âmbito da APS no Brasil, sobretudo em regiões com maior vulnerabilidade, como a tríplice fronteira — Brasil, Paraguai e Argentina^(4,11,12)^.

A necessidade de avaliar as ações para o seguimento do prematuro na APS é justificada pelo entendimento de que esses serviços precisam dispor de práticas integrais, adequadas, oportunas e baseadas em evidências científicas. A finalidade é que tais serviços contribuam com a melhoria das condições de vida e saúde e garantam o bom desenvolvimento infantil, sobretudo para as crianças que nasceram prematuras

## OBJETIVO

Validar o conteúdo do guia Qualiprematuro sobre avaliação da qualidade do seguimento de crianças nascidas prematuras no âmbito da APS.

## MÉTODOS

### Aspectos éticos

A pesquisa foi aprovada pelo Comitê de Ética em Pesquisa e respeitou todos os preceitos éticos de pesquisas que envolvem seres humanos, segundo a Resolução nº466/2012. Para manter o anonimato dos participantes, eles foram identificados com a letra J (juiz) e o número sequencial da avaliação (J1, J2…). Os juízes enfermeiros atuantes nos serviços de APS foram identificados pela letra E (enfermeiro), seguida do número da avaliação (E1, E2…). Todos os indivíduos do estudo forneceram o Consentimento Livre e Esclarecido, com a maioria o fazendo de forma on-line.

### Desenho do estudo

Este estudo metodológico utilizou a Técnica Delphi^(13)^ para validar o conteúdo do guia Qualiprematuro^(10)^. Foram seguidas as diretrizes do *Standards for Quality Improvement Reporting Excellence* (SQUIRE)^(14)^, destinadas a relatórios descritivos sobre estudos para melhorar a qualidade de saúde.

### Descrição do guia avaliativo Qualiprematuro

A primeira versão do guia Qualiprematuro^(10)^ foi elaborada em 2021 e compreendeu um questionário com 65 itens para ser respondido por profissionais de saúde (enfermeiros ou médicos) atuantes em serviços de APS. Foi organizado em cinco domínios, a fim de abranger elementos que qualificam o seguimento da saúde da criança nascida prematura, quais sejam: Planejamento da alta hospitalar e organização do plano de cuidados (11 questões); Seguimento domiciliar em visita e teleatendimento (11 questões); Seguimento da saúde infantil para promover saúde e prevenir agravos (12 questões); Integração entre serviços de saúde, educação e acompanhamento especializado (19 questões); e Apoio e suporte familiar para o cuidado (12 questões).

Foram atribuídas quatro respostas de escolha única para cada questão, com valores de 1 (Inadequado), 2 (Regular), 3 (Bom) e 4 (Excelente). No fim da avaliação de cada domínio, os valores são somados, produzindo escores que mostram como o serviço de saúde realiza o seguimento da criança pré-termo. Foi prevista a avaliação de cada domínio de forma separada, considerando que o serviço pode mostrar-se inadequado em um domínio, mas excelente em outro. Ao final, são atribuídas a cada domínio as seguintes avaliações: Inadequado, Regular, Adequado/Bom ou Excelente.

### Local do estudo e seleção dos participantes

Foram necessárias duas etapas para realizar o estudo. Na primeira, participaram juízes de estados brasileiros diversos, que foram contatados on-line pelo pesquisador. Na segunda etapa, os juízes eleitos eram enfermeiros atuantes em serviços de APS do município de Foz do Iguaçu, estado do Paraná (PR), Brasil.

A busca pelos juízes da primeira etapa foi realizada utilizando-se os filtros de pesquisa da plataforma Lattes do Conselho Nacional de Desenvolvimento Científico e Tecnológico (CNPq) - Brasil. A pesquisa também ocorreu na página dos programas de pós-graduação stricto sensu de universidades públicas brasileiras.

Nessa primeira etapa de validação, os 17 juízes incluídos eram enfermeiros com a titulação mínima de mestre (6 mestres e 11 doutores), que trabalhavam na atenção direta aos recém-nascidos e/ou cuja área de pesquisa descrita no Lattes se relacionava com a saúde do recém-nascido e da criança. Foram excluídos da pesquisa os enfermeiros que não responderam ao convite após três tentativas de contato (por e-mail ou por mensagens de textos instantâneas). Inicialmente foram enviados 50 convites para profissionais de todas as regiões brasileiras, com resposta de 17 profissionais das Regiões Sul e Sudeste, que procederam com a avaliação do conteúdo do guia avaliativo.

Na segunda etapa, os pesquisadores escolheram 20 juízes que eram enfermeiros atuantes em serviços de APS de um município brasileiro de fronteira. O critério de exclusão foi estar afastado do trabalho por licença ou férias no período de coleta de dados.

A amostra dos participantes do processo de validação de conteúdo nas duas etapas foi definida considerando outros estudos de validação de instrumentos^(15,16)^.

### Coleta de dados

A primeira rodada de envio do questionário para os juízes ocorreu em junho de 2023. Já em agosto do mesmo ano, foi enviado novamente apenas para alcançar um número representativo de participantes. A coleta de dados da segunda etapa aconteceu em setembro de 2023. Considerando a Técnica Delphi, não foram necessárias novas rodadas de avaliação após as análises estatísticas, visto que o valor de concordância proposto pelo método utilizado foi alcançado na primeira rodada.

Os juízes da primeira etapa foram convidados por meio de uma carta-convite, encaminhada via e-mail e ou por WhatsApp^®^. Na mensagem, foram explicados os objetivos da pesquisa e foi explanado que, ao aceitar convite, o participante devia assinar digitalmente o Termo de Consentimento Livre e Esclarecido (TCLE) em duas vias — uma via ficava com o juiz, e a outra era encaminhada ao remetente para ser arquivada. Em seguida, foi enviado o guia para sua avaliação e validação (via e-mail e/ou pelo Google Forms^®^). Depois de avaliado, os juízes devolveram-no de forma on-line aos pesquisadores, com as devidas recomendações de alteração do material.

Na segunda etapa, os enfermeiros participantes pertenciam a unidades de saúde diferentes, abrangendo os cinco distritos de saúde de Foz do Iguaçu, PR. Assim, foi possível verificar a clareza e a compreensibilidade do guia no município como um todo. A unidade de saúde inserida foi eleita por meio de sorteio. Em seguida, via telefone e/ou mensagem de texto, realizou-se o convite aos enfermeiros para participarem da pesquisa, sendo que, nessa etapa, todos os 20 enfermeiros assinaram o TCLE de modo on-line, pois optaram por utilizar o Google Forms^®^.

### Instrumento de coleta de dados

O instrumento utilizado para a coleta de dados com os juízes foi uma adaptação do Qualiprematuro. Foram atribuídas as seguintes interpretações para cada questão: Concordo, Concordo parcialmente e Discordo. Essas interpretações foram organizadas em uma Escala Likert, atribuindo-se valores de 1 a 3, sendo: (1) Discordo; (2) Concordo parcialmente; e (3) Concordo. Caso a interpretação do juiz fosse “Discordo” ou “Concordo parcialmente”, havia um espaço para descrever a sugestão para a reformulação da questão.

Na segunda etapa de coleta, foram atribuídas as seguintes interpretações para cada questão: Confusa, Pouco clara e Clara e de fácil compreensão. Essas interpretações foram organizadas em uma Escala Likert, atribuindo-se valores de 1 a 3, sendo: (1) Confusa, (2) Pouco clara e (3) Clara e de fácil compreensão. Também foi disponibilizado um espaço para os enfermeiros darem sugestões caso a questão recebesse a avaliação “Confusa” e/ou “Pouco clara”.

### Análise dos dados

A análise dos resultados foi realizada pelo coeficiente alfa de Cronbach. O valor mínimo aceitável para o alfa foi de 0,70, visto que valores inferiores representariam uma inconsistência ou questões inadequadas. Por sua vez, valores acima de 0,90 poderiam descrever uma redundância ou duplicação nos itens avaliados do mesmo elemento ou constructo, sendo, nesses casos, preferíveis valores de alfa entre 0,80 e 0,90^(17)^.

## RESULTADOS

### Caracterização dos juízes participantes da primeira etapa

Participaram 17 juízes, todos enfermeiros, com titulação mínima de mestre, atuantes na docência em universidades públicas e ou na prática profissional com crianças nascidas prematuras, da região Sul e Sudeste do Brasil.

### Análise de Confiabilidade do guia Qualiprematuro – primeira etapa

Para a análise de confiança interna do guia (análise de confiabilidade), as Questões 30, 40, 43, 46, 47, 50, 56, 58, 64 e 65 foram retiradas da análise, pois a resposta era a mesma (Concordo) para todos os 17 participantes.

Segundo o alfa de Cronbach, a análise total de Confiabilidade do guia mostrou correlação satisfatória das questões, com valor igual a 0,989. Considerando separadamente os cinco domínios, a análise igualmente apresentou confiabilidade satisfatória, como mostra a Tabela 1.

**Tabela 1:** Análise de confiabilidade distribuída entre os domínios do guia avaliativo Qualiprematuro. Foz do Iguaçu, Paraná, Brasil, 2023

Escalas	Alfa de Cronbach
Análise total	0.989
Domínio I	0.970
Domínio II	0.957
Domínio III	0.958
Domínio IV	0.972
Domínio V	0.965

Conforme a análise de correlação, mesmo se qualquer questão fosse retirada, o alfa de Cronbach permaneceria entre 0,989 e 0,990, o que são indicadores muito satisfatórios.

Para detalhar a avaliação do guia, identifica-se o valor do teste para cada questão, verificando a possibilidade de retirar qualquer uma das questões, caso seja necessário, tendo como base os valores de “correlação item-total”, que mede a correlação do valor do teste de todo o guia (total) com cada questão (item). Segundo a avaliação, embora não tenha sido constatada estatisticamente a necessidade, foi suprimida a Questão 6 do Domínio I com base no julgamento qualitativo dos juízes, por se mostrar redundante. Para essa questão, a correlação item-total alfa de Cronbach identificou resultado satisfatório, variando entre 0,948 a 0,974.

### Caracterização dos juízes enfermeiros da APS - segunda etapa

Foram 20 enfermeiros que participaram da etapa de verificação da clareza e compreensibilidade do conteúdo do guia, sendo 3 homens e 17 mulheres, com idades entre 29 e 47 anos. Eram 5 mestres, 2 mestrandos e 13 especialistas em diversas áreas da saúde. A maioria atuava há mais de cinco anos na APS.

### Análise de Confiabilidade do guia Qualiprematuro: clareza e compreensibilidade

Segundo o alfa de Cronbach, a análise total de Confiabilidade mostrou concordância satisfatória das questões, com escala superior de 0,993. A análise específica de confiabilidade de cada um dos cinco domínios igualmente apresentou correlação satisfatória pelo alfa de Cronbach, embora no Domínio III tenha ocorrido uma diminuição do coeficiente sem prejudicar a confiabilidade, como mostra a Tabela 2.

**Tabela 2:** Análise de Confiabilidade quanto à clareza e compreensibilidade do guia avaliativo, distribuída entre os domínios do Qualiprematuro. Foz do Iguaçu, Paraná, Brasil, 2023

Escalas	Alfa de Cronbach
Análise total	0.993
Domínio I	0.961
Domínio II	0.977
Domínio III	0.876
Domínio IV	0.984
Domínio V	0.981

Na correlação item-total, o alfa de Cronbach identificou resultados satisfatórios, variando entre 0,850 e 0,985, contudo as Questões 11, 21, 23, 46 e 63 não demonstraram boa correlação item-total. Mesmo assim, caso ocorresse a retirada de alguma dessas questões, essa ação não prejudicaria a análise da confiabilidade, como mostra a Tabela 3. Portanto, decidiu-se por mantê-las, pois avaliam questões importantes para o acompanhamento da criança prematura.

**Tabela 3:** Estatísticas da Confiabilidade do item das questões com baixa correlação item-total distribuídas nos cinco domínios. Foz do Iguaçu, Paraná, Brasil, 2023

	Se o item for eliminado	
	Correlação item-total	Alfa de Cronbach
Q11	0.513	0.965
Q21	0.688	0.980
Q23	0.196	0.883
Q46	0.546	0.985
Q63	0.667	0.984

Considerando que a análise qualitativa dos juízes determinou a retirada da Questão 6 do Domínio I por redundância, houve necessidade de readequação do guia Qualiprematuro. Essa readequação envolveu o número das questões de todos os domínios e o escore de avaliação do Domínio I, considerando que a somatória do valor de cada questão era de 11 a 44 antes, passando agora para o intervalo de 10 a 40. O Quadro 1 mostra essa readequação.

**Quadro 1:** Distribuição das questões nos cinco domínios após a readequação do guia Qualiprematuro e seus respectivos escores de avaliação. Foz do Iguaçu, Paraná, Brasil, 2023

Domínios	Questões anteriores	Questões atuais	Escores da primeira avaliação	Escores da avaliação final
I	1,2,3,4,5, 6,7,8,9,10, 11	1,2,3,4,5, 6,7,8,9,10	*Inadequado* (pontuação 11 a 21) *Regular* (pontuação 22 a 32) *Bom* (pontuação 33 a 43) *Excelente* (pontuação 44)	*Inadequado* (pontuação 10 a 19) *Regular* (pontuação 20 a 29) *Bom* (pontuação 30 a 39) *Excelente* (pontuação 40)
II	12,13,14, 15,16,17, 18,19,20, 21,22	11,12,13, 14,15,16, 17,18,19, 20,21	*Inadequado* (pontuação 11 a 21) *Regular* (pontuação 22 a 32) *Bom* (pontuação 33 a 43) *Excelente* (pontuação 44)	*Inadequado* (pontuação 11 a 21) *Regular* (pontuação 22 a 32) *Bom* (pontuação 33 a 43) *Excelente* (pontuação 44)
III	23,24,25, 26,27,28, 29,30,31, 32,33,34	22,23,24, 25,26,27, 28,29,30, 31,32,33	*Inadequado* (pontuação 12 a 23) *Regular* (pontuação 24 a 35) *Bom* (pontuação 36 a 47) *Excelente* (pontuação 48)	*Inadequado* (pontuação 12 a 23) *Regular* (pontuação 24 a 35) *Bom* (pontuação 36 a 47) *Excelente* (pontuação 48)
IV	35,36,37, 38,39,40, 41,42,43, 44,45,46, 47,48,49, 50,51,52, 53	34,35,36, 37,38,39, 40,41,42, 43,44,45, 46,47,48, 49,50,51, 52	*Inadequado* (pontuação 19 a 37) *Regular* (pontuação 38 a 56) *Bom* (pontuação 57 a 75) *Excelente* (pontuação 76)	*Inadequado* (pontuação 19 a 37) *Regular* (pontuação 38 a 56) *Bom* (pontuação 57 a 75) *Excelente* (pontuação 76)
V	54,55,56, 57,58,59, 60,61,62, 63,64,65	53,54,55, 56,57,58, 59,60,61, 62,63,64	*Inadequado* (pontuação 12 a 23) *Regular* (pontuação 24 a 35) *Bom* (pontuação 36 a 47) *Excelente* (pontuação 48)	*Inadequado* (pontuação 12 a 23) *Regular* (pontuação 24 a 35) *Bom* (pontuação 36 a 47) *Excelente* (pontuação 48)

## DISCUSSÃO

Este estudo permitiu obter uma visão detalhada do processo de validação do conteúdo do guia, cujo objetivo principal é avaliar a qualidade do seguimento de crianças nascidas prematuras no âmbito da APS. Essa ferramenta relevante para o campo da saúde teve seu conteúdo criteriosamente avaliado por juízes autores de pesquisa/com experiência na área da saúde da criança prematura. A bagagem desses profissionais refletiu no compromisso de garantir uma avaliação precisa e específica do guia. Além disso, foi realizada uma análise da clareza de seu conteúdo por profissionais de saúde (enfermeiros) atuantes na APS de um município brasileiro de tríplice fronteira — Brasil, Paraguai e Argentina.

A inclusão desses juízes demonstrou a relevância de considerar a realidade local ao avaliar a eficácia do seguimento da criança pré-termo na APS, garantindo uma avaliação da efetividade do guia^(15,16)^. Com isso, pretendeu-se garantir uma importante representação do conhecimento e da experiência necessários à avaliação do Qualiprematuro.

A participação dos profissionais da APS foi de grande valia, haja vista a necessidade de dar maior clareza ao guia. Isso assegura a assistência integral após a alta hospitalar, considerando o maior risco de adoecimento e possíveis alterações no crescimento e desenvolvimento^(7,12)^. Vale apontar que, mesmo com os avanços do cuidado longitudinal na APS, ainda é visível a fragilidade em lidar com as causas evitáveis de mortalidade e sensíveis no acompanhamento da saúde, sobretudo para grupos vulneráveis, como as crianças nascidas prematuras.

A análise da validação do conteúdo do Qualiprematuro visou aprimorar o guia e fornecer um suporte prático para melhorar a eficácia dos serviços de APS^(10)^. A inclusão de áreas fronteiriças para a análise destacou a necessidade de considerar diferentes contextos geográficos ao planejar intervenções para crianças pré-termo, promovendo, assim, uma abordagem mais específica ao contexto^(4,11)^. Essa estratégia objetivou melhorar a validade e a confiabilidade dos resultados, contribuindo para a qualidade metodológica desse guia avaliativo.

A escolha pela Técnica Delphi foi importante para construir um guia sólido e confiável. Trata-se de uma ferramenta reconhecida por estruturar e validar informações por meio do consenso de especialistas^(18)^. Seu uso foi fundamental para avaliar itens que reportam a qualidade da vigilância da saúde dos prematuros na APS, preenchendo uma lacuna na literatura científica sobre a avaliação desses cuidados específicos^(10)^. A opção por duas rodadas de avaliação evidenciou uma metodologia cuidadosa, indicando a consistência e a representatividade dos dados coletados.

A escala Likert para a coleta de dados, com interpretações que variam de “Discordo” a “Concordo”, viabilizou uma abordagem quantitativa, crucial para a análise estatística subsequente. Essa técnica tem sido amplamente utilizada em estudos sociais em que são coletadas percepções não quantitativas sobre um tema específico^(19)^. Além disso, incluir espaços para sugestões de reformulação proporcionou uma dimensão qualitativa, possibilitando que os juízes expressassem opiniões detalhadas sobre cada questão do guia.

A análise dos resultados foi por meio do coeficiente alfa de Cronbach^(20)^, o qual mostrou valor geral satisfatório na avaliação dos dois grupos de juízes (pesquisadores/profissionais e enfermeiros da APS), ou seja, 0,989 e 0,993, respectivamente. Do mesmo modo, os resultados foram satisfatórios na avaliação distribuída nos cinco domínios. É fundamental ter ciência de que o valor de alfa é afetado pelo número de itens da escala. À medida que esse número aumenta, a variância é cada vez mais incorporada sistematicamente ao numerador, levando a estimativas inflacionadas da consistência da escala^(21,22)^. Além disso, deve-se levar em conta que, se o tamanho da amostra não for considerado, o valor do alfa de Cronbach também pode ser inflacionado^(23,24)^.

Estudos que compararam instrumentos de validação utilizando o alfa de Cronbach mostraram que a intensidade da correlação entre os itens de um questionário pode ser avaliada mediante a exclusão (como no presente estudo) de um item da escala de medição com resultados igualmente satisfatórios, independentemente da questão a ser removida^(25,26)^. Se o coeficiente alfa aumenta, sugere-se que o item removido não tem alta correlação com os demais itens do questionário. Caso o coeficiente diminua, infere-se que esse item está altamente correlacionado com os demais da escala. Assim, o alfa de Cronbach foi um indicador importante para avaliar a confiabilidade do guia, revelando como cada item contribuiu para essa confiabilidade, variando de 0,949 a 0,976 para os juízes e de 0,850 a 0,985 para os enfermeiros da APS.

A proposta do guia de avaliar a saúde da criança prematura foi abrangente e bem estruturada. Inicialmente continha 65 itens. Após as recomendações qualitativas dos participantes, uma questão do Domínio I foi retirada, por ser redundante. Na sequência, o guia foi organizado em 64 itens, todos os quais com o objetivo de revelar nuances importantes do cuidado dos profissionais (sobretudo médicos e enfermeiros) e da atenção prestada pela APS^(10)^.

A organização em cinco domínios permaneceu sem quaisquer alterações. Eles incluíam o planejamento da alta hospitalar, acompanhamento domiciliar da criança, saúde infantil, integração de serviços e apoio familiar. Essa organização abrangeu integralmente as condições de acompanhamento da saúde dessas crianças. Tal diversidade de áreas reflete a complexidade do acompanhamento das crianças pré-termo, desde o planejamento da alta até o essencial apoio familiar. A aplicação das respostas avaliadas, uma por questão, objetivou fornecer uma avaliação detalhada e criteriosa da eficácia baseada em evidências científicas, a fim de qualificar o seguimento da criança prematura^(10,27)^.

Como o presente estudo realizou as etapas de validação de conteúdo, a soma dos valores ao final de cada domínio proposto forneceu uma métrica importante para analisar a atenção prestada. Avaliar cada área individualmente, reconhecendo que um serviço pode ter pontos fortes, mas necessitar de melhorias em outros, incentiva e motiva as equipes de saúde e os serviços de modo geral a buscarem aperfeiçoamento para a prática e estruturação do serviço.

A validação de conteúdo de um instrumento possibilita que a ferramenta meça o que afirma medir^(28)^. Isso é fundamental para garantir que os resultados sejam confiáveis e possam ser utilizados com segurança na tomada de decisões. Tal validação ajuda a identificar e corrigir possíveis deficiências da ferramenta, garantindo precisão nas respostas, o que é vital sobretudo quando se trata de informações sensíveis, as quais podem ter consequências graves.

É essencial destacar que a validade está intrinsicamente relacionada à confiabilidade do instrumento. Em outras palavras, a validade pode ser compreendida como o grau de associação entre os escores de um teste e algum critério externo do mesmo teste, tais como os resultados obtidos em outra avaliação, definições conceituais ou formulação de objetivos^(29,30)^.

O processo de validação determina a confiabilidade inicial da ferramenta e fornece informações para a sua melhoria contínua. Pode-se afirmar que a avaliação da qualidade do seguimento da saúde da criança prematura beneficia os indivíduos em questão e contribui para aprimorar a eficiência e a eficácia dos serviços prestados na APS.

Portanto, para o cuidado e seguimento da criança prematura, a literatura ainda se encontra incipiente. Desse modo, a proposta da validação do conteúdo do Qualiprematuro, por ora em região de fronteira, consegue avaliar um tipo de contexto cultural e reforça a importância de ferramentas para amparar e avaliar as ações e práticas nos serviços de seguimento da saúde.

Este estudo ofereceu um olhar crítico sobre as práticas atuais, apontando para possíveis áreas de aprimoramento na abordagem da saúde da criança pré-termo no contexto da APS. A abordagem específica para regiões fronteiriças, cenário de trabalho dos enfermeiros da APS deste estudo, reconhece as vulnerabilidades desses territórios e mira em estratégias para otimizar o seguimento da saúde nesses locais^(4,11,12)^.

Adicionalmente, a Técnica Delphi aplicada on-line possibilitou a participação de juízes de diversas localidades, garantindo diferentes experiências para a validação do guia. A relevância em abordar questões específicas de uma diversidade regional realça a importância de considerar contextos geográficos únicos no desenvolvimento de estratégias de vigilância e monitoramento. A análise dos dados foi realizada de forma iterativa, alcançando um valor consistente proposto pelo método estatístico. Espera-se que esses resultados inspirem iniciativas futuras e incentivem pesquisas para monitorar a saúde de bebês pré-termo, fornecendo uma base sólida para intervenções oportunas e eficazes.

O investimento na saúde dessas crianças deve estender-se para além do período da hospitalização, pois cada uma delas tem potencial para superar os desafios iniciais da vida e tornar-se um membro saudável, contribuindo com a sociedade. Portanto, prestar atenção à saúde de crianças nascidas prematuras garante um começo saudável e representa um compromisso significativo para a construção de uma comunidade segura e sustentável^(1,2)^.

## Limitações do estudo

No processo de validação do conteúdo do Qualiprematuro, não foi executada a etapa de pré-teste e ou pesquisa-piloto, a qual deverá ocorrer em pesquisas futuras. Além disso, será relevante incluir outros profissionais da APS responsáveis pelo seguimento das crianças nascidas prematuras, já que este estudo limitou-se a incluir enfermeiros.

## Contribuições para a área da enfermagem

A validação do conteúdo do Qualiprematuro forneceu dados importantes, padronizados e culturalmente sensíveis. Assim, torna-se uma ferramenta apropriada para a formação de profissionais de saúde, sobretudo de enfermeiros, na prestação de um apoio eficaz e personalizado a essas crianças e suas famílias. As contribuições ultrapassam a mensuração de resultados, alcançando a prática da enfermagem na investigação e na promoção de políticas de saúde que visam ao desenvolvimento global e bem-estar de crianças nascidas prematuras.

Ao fornecer informações sobre a qualidade da vigilância de bebês pré-termo, espera-se contribuir para uma compreensão mais ampla da prática de saúde na Atenção Primária, particularmente em regiões consideradas vulneráveis, como a faixa de fronteira. Tal contribuição se alinha com os Objetivos de Desenvolvimento Sustentável e da Estratégia Mundial para a Saúde das Mulheres, Crianças e Adolescentes, implicando a adoção de abordagens mais integradas e colaborativas para combater as desigualdades sociais, ambientais e econômicas.

O conteúdo do guia Qualiprematuro proporciona melhoria na qualidade do seguimento da saúde da criança pré-termo, pois garante que essas crianças recebam a atenção e os cuidados adequados na APS. Além disso, padroniza e direciona a prática clínica com diretrizes e recomendações validadas. Como desdobramento, ainda pode ser uma ferramenta para aprimorar a comunicação da equipe e a eficácia dos serviços de saúde, beneficiando, assim, a coordenação do cuidado da criança prematura.

## CONCLUSÕES

O conteúdo do guia avaliativo Qualiprematuro foi considerado validado. Nesse processo, foi possível evidenciar sua representatividade no que se refere à compreensão teórica e prática do cuidado. Esta foi considerada uma etapa importante no desenvolvimento do guia, haja vista a constatação de que o material é confiável e conciso naquilo que pretende medir.

A metodologia, adotando a Técnica Delphi, permitiu construir consenso entre especialistas — juízes com experiência na saúde neonatal e em pesquisas — e enfermeiros da prática profissional na APS. Deve-se mencionar o caráter iterativo da análise dos dados por meio de escores satisfatórios do coeficiente alfa de Cronbach nas duas etapas de avaliação e na análise da distribuição entre os domínios, garantindo a confiabilidade, validade e clareza dos resultados. Importante destacar que, mesmo com índices favoráveis, todas as recomendações dos especialistas foram consideradas para a construção final do guia, já que, inicialmente, o guia contava com 11 questões no Domínio I, passando a 10 após a readequação. No total, a versão inicial tinha 65 questões, enquanto a versão final apresentou 64 questões.

Assim, a validação do conteúdo do Qualiprematuro, evidenciado como uma ferramenta de avaliação inovadora e com metodologia abrangente, contribuirá para a compreensão teórica dos cuidados e recomendará práticas importantes para os profissionais de saúde quanto ao seguimento da saúde da criança prematura. O compromisso do estudo com a transparência, consistência e rigor metodológico fortalece futuras iniciativas e pesquisas focadas na vigilância da saúde de crianças nascidas prematuras, respondendo à necessidade de melhorar as práticas e os resultados de saúde para essa população em todo o mundo.
